# Symmetry breaking of paracrystalline topology in amorphous silicon

**DOI:** 10.1038/s41598-025-15737-8

**Published:** 2025-08-27

**Authors:** Koji S. Nakayama, Masahiko Nishijima, Yicheng Zhang, Koji Inoue, Chuantong Chen, Minoru Ueshima, Katsuaki Suganuma

**Affiliations:** 1 The Institute of Scientific and Industrial Research, The University of Osaka, 8-1 Mihogaoka, Ibaraki, 567-0047 Osaka Japan; 2 International Research Center for Nuclear Materials Science, Institute for Materials Research, Tohoku University, 2145-2, Oarai, Ibaraki, 311-1313 Japan; 3 Daicel Corporation, 2-18-1, Konan, Minato-ku, Tokyo, 108-8230 Japan

**Keywords:** Engineering, Materials science, Nanoscience and technology

## Abstract

The atomic structure of amorphous Si (a-Si) has traditionally been described by the continuous random network (CRN) model, which consists of the four-coordinated Si with a non-periodic structure. However, the paracrystalline model, consisting of strained nanocrystals embedded within a disordered matrix, has gained traction. This shift is largely driven by fluctuation electron microscopy observations, which reveal the distinct diffraction patterns that are inconsistent with the CRN model. However, the degree and nature of paracrystallinity remain unclear due to a lack of experimental approaches capable of revealing finite size effects. In this paper, we present the atomic structure of a-Si that appeared in a liquid quenched Ag-Si alloy. Fast Fourier transform and electron diffraction patterns exhibit excellent agreement with molecular dynamics simulations. Furthermore, nano-beam electron diffraction reveals distinct diffraction spots that support the paracrystalline model. Importantly, these diffraction spots violate the conventional crystallographic extinction rule, implying symmetry breaking within the paracrystalline structure. This is significant because the appearance of forbidden reflections offers direct evidence of local structural changes and provides new insight into the underlying disorder in a-Si.

## Introduction

Thin films a-Si have been conventionally produced by vacuum evaporation^1–4^, chemical vapor deposition^5,6^, ion implantation^7–9^, and electron beam irradiation^10,11^. Meanwhile, the formation of bulk a-Si that solidifies directly from the melt was predicted based on the classical nucleation theory when the free energy change of a-Si that becomes lower than that of liquid Si when the liquid Si is supercooled by 245 K^12^. This was supported by first-principles molecular dynamics (MD) simulations, in which the liquid-liquid phase transition occurs from the high-density liquid to the low-density liquid in the deeply supercooled region^13–15^. This phase transition to the low-density liquid would lead to the solidification of a-Si. However, all experimental attempts to obtain bulk a-Si by melt quenching techniques have failed^12^. Recently, Nakayama et al. reported the formation of a-Si in the liquid quenched Ag-Si alloy, where the eutectic reaction leads to the nanoscale phase separation between Ag and Si, and the phase separated Si is significantly supercooled. This results in the amorphization of Si^16^. These findings indicate that a-Si can be produced directly from the melt, opening up the possibility of bulk a-Si production.

The atomic structure of thin film a-Si has been characterized by neutron diffraction^3,4^, X-ray diffraction^2,9^, and transmission electron microscopy (TEM)^8,11^, and has traditionally been described by the CRN model^17–21^. The ideal CRN model of a-Si is characterized by minimal deviation from the four-fold coordination with non-periodic structure, which is metastable with respect to crystalline silicon (c-Si). The radial distribution function (RDF) derived from the diffraction experiments^22^ has been used to interpret the CRN model. However, this RDF approach is insensitive to the local inhomogeneities of a-Si. For example, selected area electron diffraction of TEM has been obtained with a minimum size of 1 μm, which provides a homogeneous distribution of structural states from an averaged volume. Meanwhile, fluctuation electron microscopy (FEM), which follows the statics of spatially resolved coherent diffraction, confirms the presence of paracrystalline order in the medium range below ~ 20 Å^21,23–25^. These findings have been supported by recent machine-learning driven MD simulations^26^.

In this study, the first question to be described is the solubility of Ag in the Si matrix. The results of scanning TEM (STEM) with the energy dispersive X-ray spectroscopy (EDS) and atom probe tomography (APT) indicate that the distribution of Ag in the Si domain is negligible and that the phase separated a-Si consists of pure Si. The second question is whether the classical MD simulations can address the atomic structures of the liquid quenched a-Si produced by the eutectic reaction in the Ag-Si alloy system. The Fourier transform pattern of the MD model shows excellent agreement with the electron diffraction with an electron beam size of ~ 20 nm. In this case, the first halo ring coincides with the 111 reflection of c-Si that is broadened by disorder. Finally, we show the results of the nano-beam electron diffraction (NBED), which allows for the acquisition of two-dimensional diffraction patterns associated with the nearest-neighbor atomic configuration^27,28^. By reducing the electron beam size below 1 nm, the distinct diffraction spots appear, supporting the paracrystalline model of a-Si. However, we find that the NBED spots do not follow the extinction rule in the diffraction principle. This is significant because the appearance of forbidden reflections reveals symmetry breaking in the paracrystals of a-Si.

## Results and discussion

### TEM observations

**Fig. 1:**
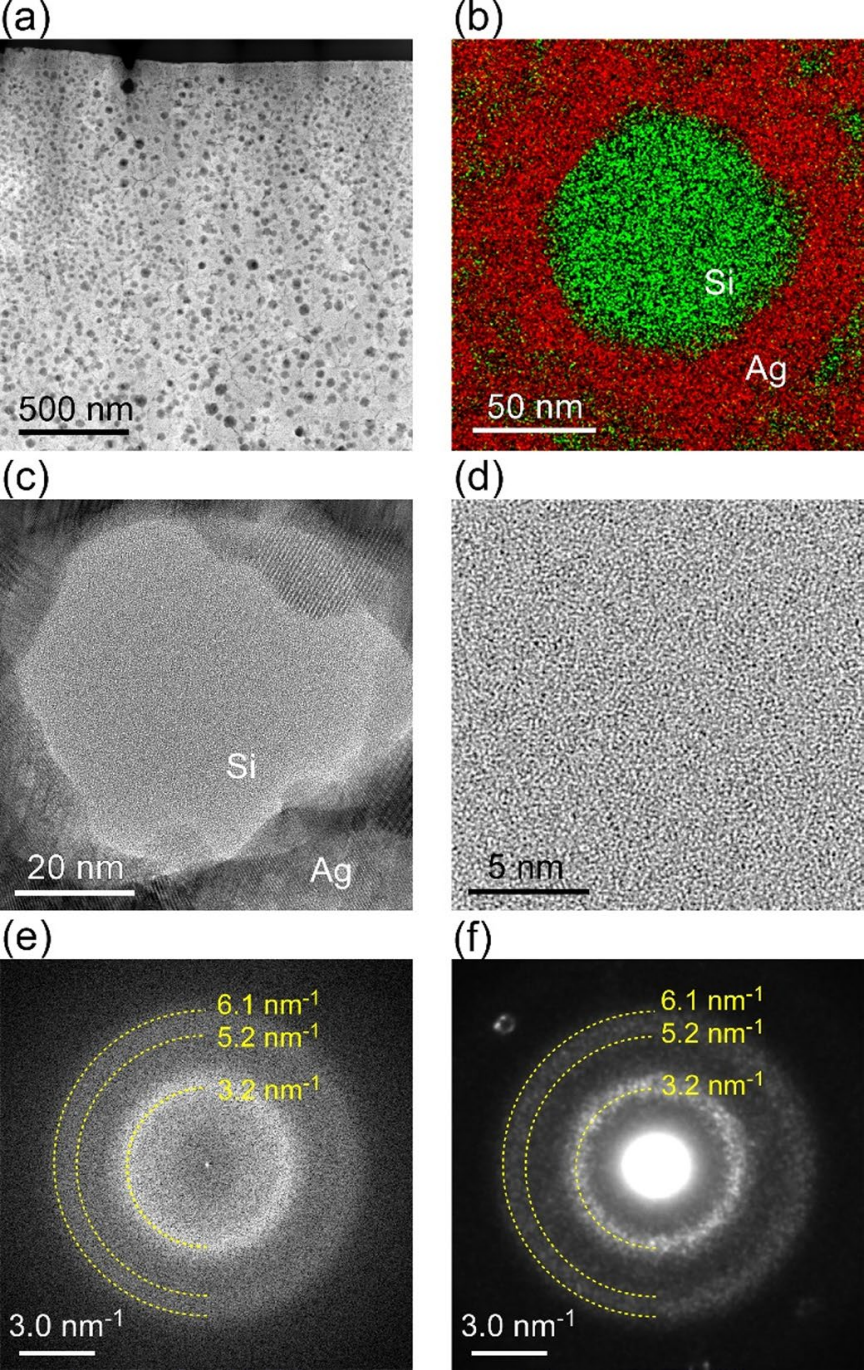
STEM-EDS and TEM observations. (a) A low magnification HAADF-STEM image for the liquid quenched Ag_79_Si_21_ (at%) alloy ribbon. The dark dots are homogeneously distributed. (b) The EDS map shows that the circular shape corresponds to (green) Si, which is surrounded by (red) Ag. (c) The bright-field TEM image for a typical circular shaped Si. (d) The image indicates a maze-like disordered atomic structure. (e) The FFT pattern obtained from the maze-like region of a-Si. The yellow dashed lines are the Debye-Scherrer rings to guide the eyes. (f) The diffraction pattern obtained from the a-Si region with a beam size of ~ 20 nm.

Figure 1(a) shows the high-angle annular dark-field (HAADF)-STEM image at low magnification. The dark dots are homogeneously distributed throughout the imaged area that is obtained from near the wheel side (see methods). The intensity of the scattered electrons from heavy atoms is stronger in the HAADF mode, thus, the brighter area corresponds to Ag and the dark dots correspond to Si. This is confirmed by the EDS map, as shown in Fig. 1(b), where a circular shape of Si (green) with a diameter of about 90 nm is surrounded by Ag (red). It is also evident that Si is distributed in the Ag matrix, but Ag is not distributed in the Si domain. This indicates the existence of a supersaturated Ag solid solution and the purity of Si, which is confirmed by the APT results (discussed later).

Figure 1(c) shows the high-resolution TEM image of a typical circular shaped Si. Figure 1(d) shows a zoomed-in image of the Si domain. A homogeneous maze-like pattern, which is typical of an amorphous structure can be observed. Figure 1(e) shows the fast Fourier transform (FFT) pattern, which is calculated from the maze-like region in Fig. 1(d). The yellow dashed lines marked in the image represent Debye-Scherrer rings. A strong ring appears at Q = 3.2 nm^− 1^ and a very weak ring appears between Q = 5.2 nm^− 1^ and Q = 6.1 nm^− 1^, where Q is the magnitude of scattering vector ($\:\left|\varvec{Q}\right|=\text{Q}=2\text{sin}\theta\:/\lambda\:$, where $\:\theta\:$ is the scattering angle and λ is the electron wavelength). Figure 1(f) shows the electron diffraction pattern obtained from the a-Si region with a beam size of ~ 20 nm. The information observed is the same as that in Fig. 1(e). However, the weak ring that appeared between Q = 5.2 nm^−1^ and Q = 6.1 nm^− 1^ is more evident. We note that the halo rings, particularly at Q = 3.2 nm^− 1^, consists of the aggregation of small diffraction spots.

### APT observations

**Fig. 2:**
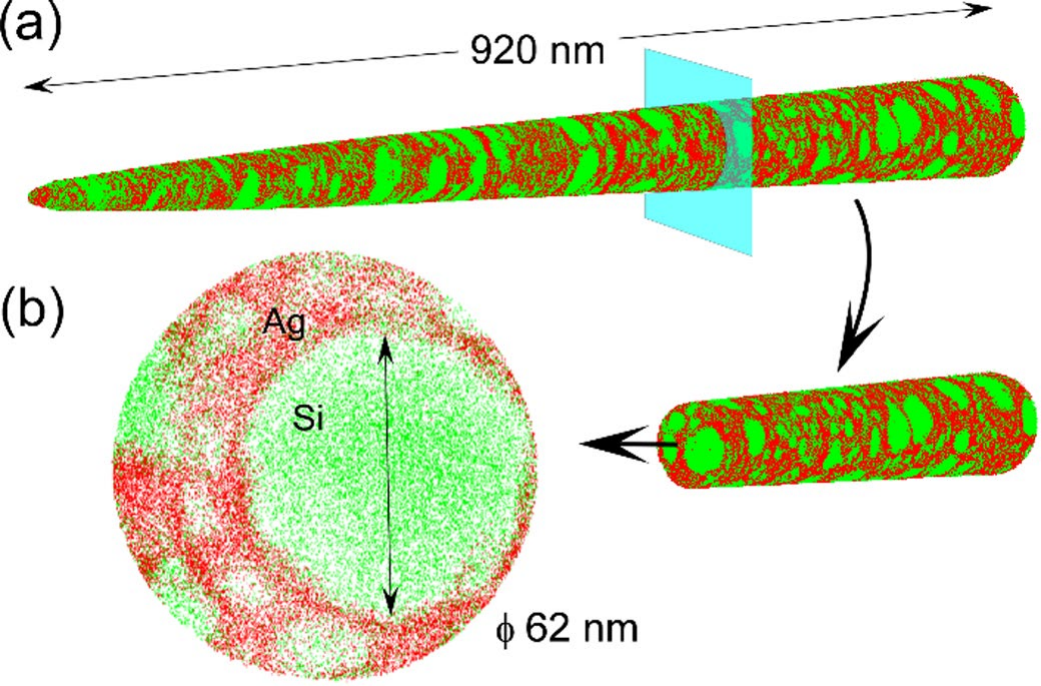
APT observations. (a) The needle shape with a length of 920 nm of the liquid quenched Ag-Si alloy is projected. It represents the phase separation of Ag and Si at the nanoscale. (b) The high magnification of the needle cross section shows that the Si domain is a spherical shape with a diameter of 62 nm.

The EDS map in Fig. 1(b) shows that the distribution of Ag within the Si domain is negligible. However, this may be an underestimation because the size of the Si domains is smaller than the thickness of the TEM sample (~ 100 nm). Therefore, there should be an overlap between Si and Ag, which could cause errors in the chemical analysis. To clarify the chemical composition of the nanoscale objects, we have conducted the APT observations. Figure 2(a) shows the APT map projected from an area about 2 μm away from the wheel side of the alloy ribbon. This sampling area is similar to the one in Fig. 1(a). The three dimensional map clearly represents the phase separation between Ag and Si, consistent with the TEM observations. Figure 2(b) shows a high-magnification cross-section of the needle. It reveals that the Si domain is spherical with a diameter of 62 nm. This confirms that the dark dots observed in the STEM image in Fig. 1(a) correspond to Si spheres. We have analyzed about 50 different points and found that the Ag concentration in the Si domain is less than 1%. Therefore, we conclude that the dissolution of Ag in Si is negligible and the composition of a-Si is pure Si. We have also detected 0.02 at% boron, which may be present in Si as a dopant.

### MD simulations

**Fig. 3:**
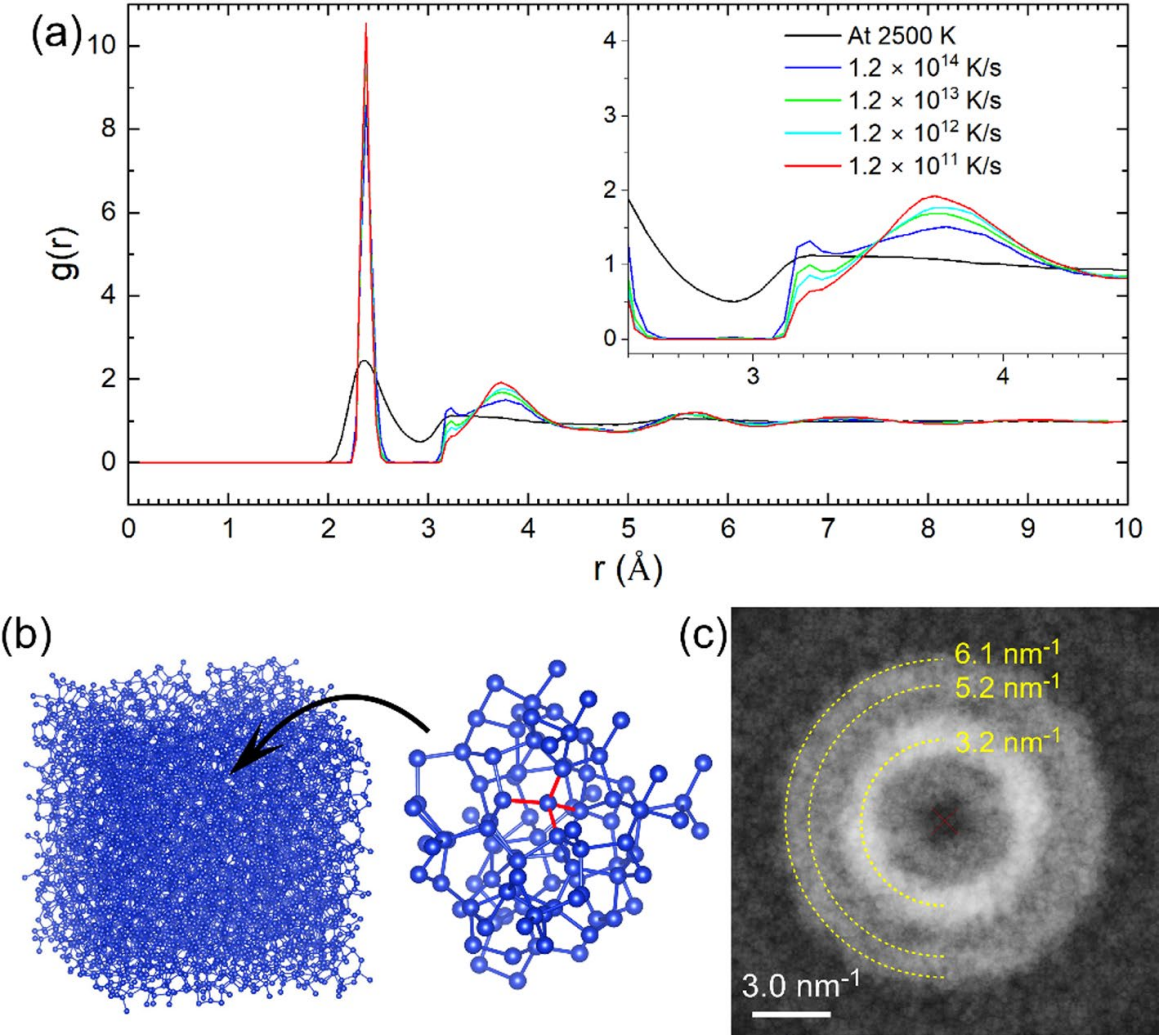
MD simulations. (a) The radial distribution function $\:g\left(r\right)$ calculated by the MD simulations with varying cooling speeds. The inset shows a magnification ranging from 2.5 Å to 4.5 Å. The shoulder peak at 3.23 Å weakens as the cooling speed decreases. (b) The atomic structure of a-Si obtained by the MD simulations with 4,096 atoms. Local tetrahedral Si bonds in the short-range order are colored red in the atomic model. (c) The Fourier transform pattern obtained from the MD model. Two halo rings are visible, where a strong ring appears at Q = 3.2 nm ^− 1^ and a weak ring appears between Q = 5.2 and 6.1 nm^− 1^.

To compare the atomic structures through the experiments, we conducted classical MD simulations using the Large-scale Atomic/Molecular Massively Parallel Simulator (LAMMPS, NanoLabo, Advanced Corp.). The simulation size was 8 unit cells with periodic boundary conditions, and 4,096 Si atoms were included in the cell. We ran a melt quenching algorithm under a canonical ensemble with Tersoff potential. The system temperature was controlled with a time step of 0.5 fs. First, the initial diamond Si structure was melted by increasing the temperature from 100 to 2500 K at a rate of 1.2 × 10^13^ K/s. To verify the MD simulations, we varied the cooling speeds after the equilibrium at 2500 K for 100 ps. Then, the system was cooled to 100 K at rates of 1.2 × 10^11^, 1.2 × 10^12^, 1.2 × 10^13^, and 1.2 × 10^14^ K/s. Figure 3(a) shows that the RDF results with the variation of the cooling speeds. The peak corresponding to the first nearest-neighbor distance appears at 2.38 Å. The second nearest-neighbor distance appears at 3.73 Å, accompanied by a shoulder at 3.23 Å. Furthermore, a small peak appears at 5.58 Å. The shoulder peak becomes weakened as the cooling speed decreases from 1.2 × 10^14^ K/s to 1.2 × 10^11^ K/s. Previous MD simulations using the Stillinger-Weber potential also indicated the shoulder peak at 3.23 Å, which depends on the cooling speed^29^. We also calculated the equilibrium state at 2500 K for 2 ns and found that the 3.23 Å peak became much broader, as shown in Fig. 3(a). This feature also agrees with the previous MD simulations using empirical potentials for the Si melt. They indicated that the shoulder peak is related to the covalent bonds in metallic states of Si^30^.

Our MD simulations are in good agreement with the previous calculations^29,30^ and effectively capture the amorphous structure of Si. Figure 3(b) shows the atomic model of a-Si obtained from the MD simulations. The model is constructed from 4096 atoms and visualized using the VESTA program^31^. The local tetrahedral configuration is highlighted by red bonds. To compare the simulation results with experimental data, we employed the ReciPro program^32^ to quantitatively simulate diffraction patterns. Figure 3(c) shows the Fourier transform image of the MD model, calculated under a cooling speed of 1.2 × 10^11^ K/s. The yellow dashed lines indicate Debye-Scherrer rings at Q = 3.2, 5.2, and 6.1 nm^− 1^. The simulated pattern closely matches both the FFT pattern in Fig. 1(e) and the electron diffraction pattern in Fig. 1(f). These results demonstrate that the experimental diffraction features of a-Si obtained from the liquid quenched Ag-Si alloy can be reliably reproduced by the classical MD simulations.

### Appearance of forbidden reflections

**Fig. 4:**
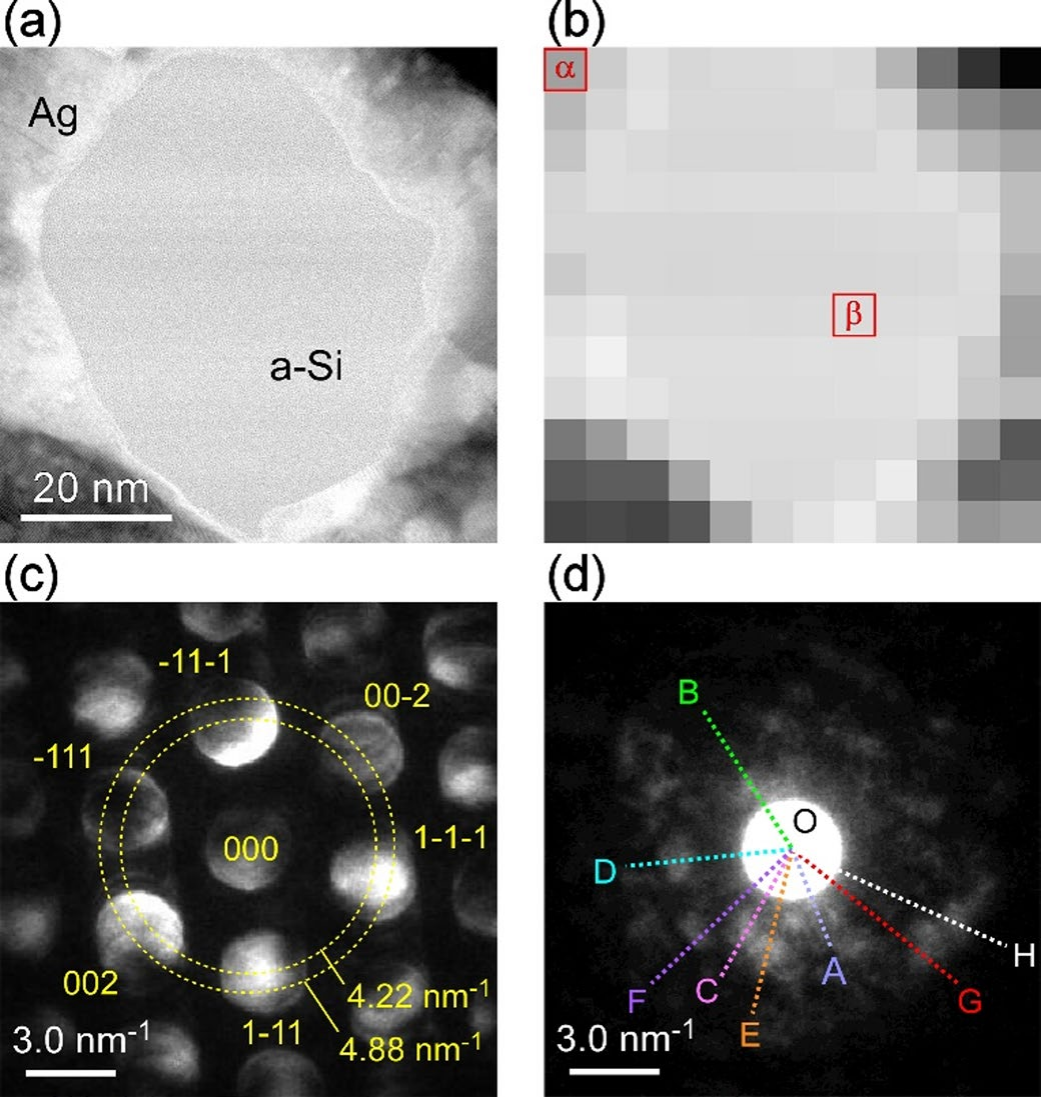
NBED results from Ag and a-Si regions. (a) A bright-field STEM image of the area resembles that in Fig. 1(c). The central domain corresponds to a-Si, which is surrounded by crystalline Ag. (b) The imaged region is divided into 12 × 12 domains. The NBED patterns are obtained from each region with a beam size below 1 nm. (c) The NBED pattern obtained from the α region in (b). The spots correspond to the face-centered cubic structure of Ag. (d) The NBED pattern obtained from the β region, which corresponds to a-Si. Distinct diffraction spots appear. Intensity profiles of the NBED patterns are measured along the lines A-H (see Fig. 5(b)).

Figure 4(a) represents a bright-field STEM image, and the observed area resembles that in Fig. 1(c). The central domain corresponds to a-Si and is surrounded by crystalline Ag. The imaged region was divided into 12 × 12 grid, as shown in Fig. 4(b) and the NBED patterns were acquired from each region. Figure 4(c) shows the NBED pattern obtained from the α region in Fig. 4(b), which corresponds to the Ag face-centered cubic structure. Figure 4(d) shows the NBED pattern obtained from the β region in Fig. 4(b). The presence of distinct diffraction spots reveals the local atomic configuration when the electron beam size is reduced below 1 nm.

The NBED patterns are analogous to the single crystal diffraction pattern^27,28^. According to the diffraction principle, the intensity of electron scattering can be expressed as^33^,

$$I\left( {\varvec{Q}} \right) \propto {\left| {F\left( {\varvec{Q}} \right)} \right|^2},$$

$F\left( {\varvec{Q}} \right)$ is the crystal structural factor, which can be written as,

$$F\left( {\varvec{Q}} \right)=\smallint \rho \left( {\varvec{r}} \right)~exp\left( {2\pi i{\varvec{Q}} \cdot {\varvec{r}}} \right)d{\varvec{r}},$$

where $\rho \left( {\varvec{r}} \right)$ is the electron density at the distance ***r***.

The structural factor for the *h*, *k*, *l* reciprocal lattice point can be written as,

$${F_{hkl}}=\mathop \sum \limits_{i} {f_i}~exp\left\{ {2\pi i\left( {h{x_i}+k{y_i}+l{z_i}} \right)} \right\},$$

where *x*_*i*_, *y*_*i*_, *z*_*i*_ are the fractional coordinates of the atom, *f*_*i*_ is the atomic scattering factor of a crystal. Using the diamond Si coordination of 8 atoms in the unit cell, we obtain.

$${F_{hkl}}={f_{Si}}\left[ {1+exp\left\{ {\pi i\left( {k+l} \right)} \right\}+exp\left\{ {\pi i\left( {l+h} \right)} \right\}+exp\left\{ {\pi i\left( {h+k} \right)} \right\}} \right]\left[ {1+exp\left\{ {\frac{\pi }{2}i\left( {h+k+l} \right)} \right\}} \right],$$

where *f*_*Si*_ is the atomic scattering factor of Si.

Thus, ${F_{hkl}}=0$ when *h*, *k*, and *l* are a mixture of even and odd numbers, or $h+k+l~=4n+2$, where n is an integer (i.e., $h+k+l~=2,~6,~10,~14 \ldots$ are forbidden)^34^.

**Fig. 5:**
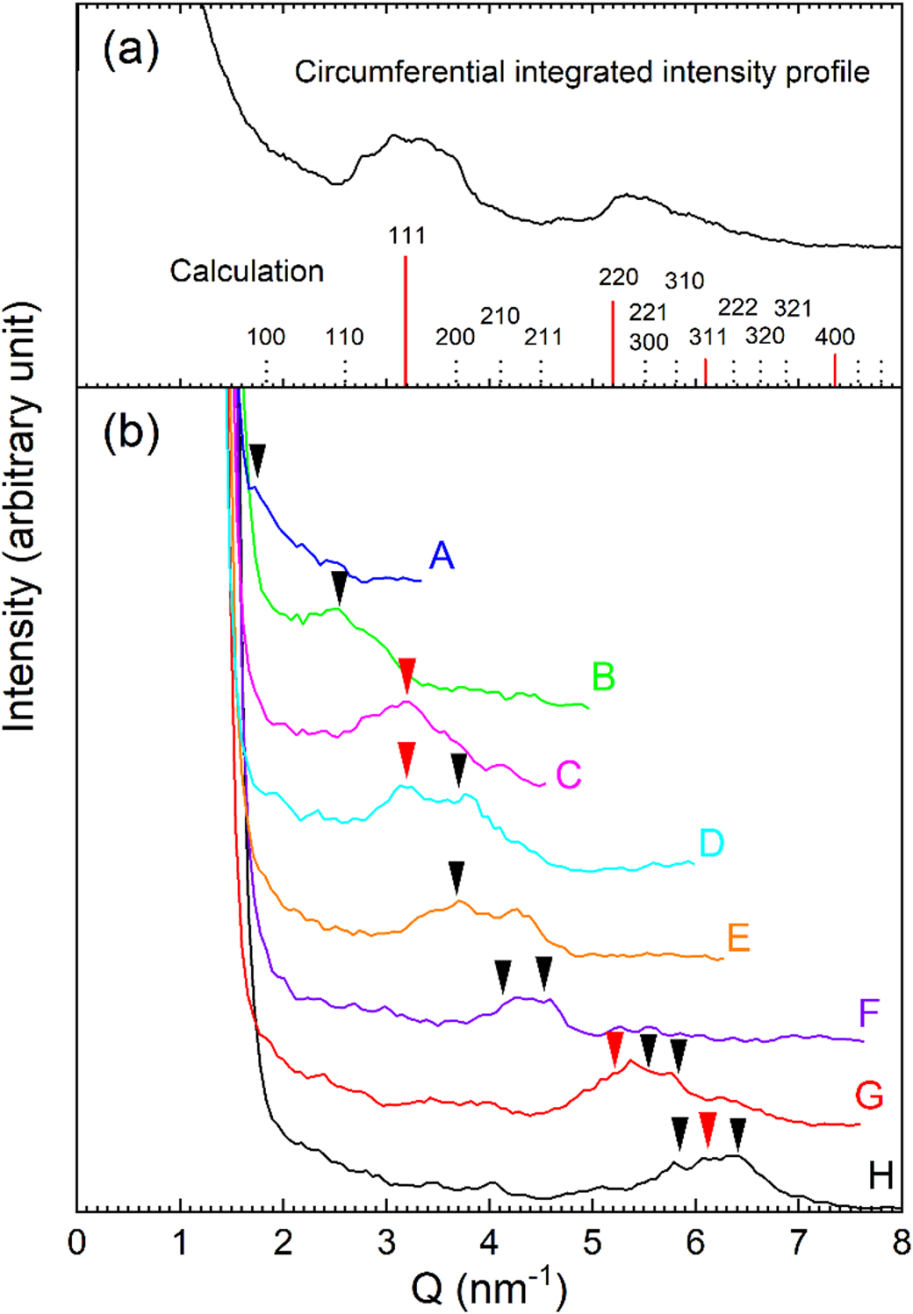
Intensity profiles of diffraction patterns. (a) The red vertical lines represent the expected Bragg peaks of c-Si for *F*_*hkl*_ ≠ 0, while the dashed vertical lines correspond to the NFR positions for *F*_*hkl*_ = 0. The solid line corresponds to the experimental intensity profile is obtained through circumferential integration of the diffraction pattern in Fig. 1(f). The first broad peak appears at Q = 3.2 nm^− 1^ corresponding to the c-Si 111 reflection. The second broad peak falls between Q = 5.2 nm^− 1^ (c-Si 220) and Q = 6.1 nm^− 1^ (c-Si 311). (b) The intensity line profiles obtained from the lines A-H in Fig. 4(d). The red arrows indicate c-Si reflections and the black arrows indicate NFRs.

Figure 5(a) represents that the red vertical lines correspond to the expected Bragg peaks c-Si for ${F_{hkl}} \ne 0$. It shows the c-Si 111, 220, 311, and 400 reflections. The intensity profile in Fig. 5(a) is the experimental one obtained by the circumferential integration of the electron diffraction pattern in Fig. 1(f). The first peak appears at Q = 3.2 nm^−1^, corresponding to the c-Si 111 reflection, is broadened by disorder, and that the second peak falls between the Q = 5.2 nm^− 1^ (c-Si 220 reflection) and Q = 6.1 nm^− 1^ (c-Si 311 reflection) peaks. Therefore, the peak positions are associated with the local tetrahedral Si bonding in the short-range order, as depicted by the red bonds of the atomic model in Fig. 3(b).

The dashed vertical lines in Fig. 5(a) correspond to the forbidden reflections, which should not appear in perfect crystals due to $\:{F}_{hkl}=0$. However, this is not the case for the paracrystals in a-Si. Figure 5(b) shows the intensity line profiles obtained from the NBED image in Fig. 4(d). A weak peak appears on the line A at Q = 1.8 nm^−1^ corresponding to the 100 nominally-forbidden reflection (NFR). The distinct peak appears on the line B at Q = 2.6 nm^− 1^ corresponding to the 110 NFR. The distinct single peak also appears on the line C at Q = 3.2 nm^− 1^ corresponding to the c-Si 111 reflection, as seen in Fig. 5(a). The twin peaks appear on the line D, where one is at Q = 3.2 nm^− 1^ and the other is at Q = 3.7 nm^− 1^. The latter corresponds to the 200 NFR. The broader 200 NFR appears on the line E, which has a shoulder peak. The shoulder would correspond to the overlap between the 210 and 211 NFRs. The shoulder peak turns into a broader peak on the line F. One is at Q = 4.1 nm^− 1^ corresponding to the 210 NFR and the other is at Q = 4.5 nm^− 1^ corresponding to the 211 NFR. A much broader peak appears on the line G, which is a combination of Q = 5.2, 5.5, and 5.8 nm^− 1^, corresponding to the c-Si 220 reflection, the 221 (or 300) NFR, and the 310 NFR, respectively. Another broader peak appears on the line H, which is a combination of Q = 5.8, 6.1, and 6.4 nm^− 1^, corresponding to the 310 NFR, the c-Si 311 reflection, and the 222 NFR, respectively. Furthermore, we have analyzed over 10 points equivalent to the β region in Fig. 4(b) in the NBED data and obtained the similar appearance of forbidden reflections, as shown in Fig. 4(d).

The appearance of c-Si 111, 220, and 311 reflections, as indicated by the red arrows in Fig. 5(a), reveals the paracrystalline topology in a-Si. More importantly, the NBED intensity profiles clearly demonstrate the peaks of the NFDs, as indicated by the black arrows in Fig. 5(b). The appearance of the forbidden reflections despite $\:{F}_{hkl}=0$ reveals the symmetry breaking in the local paracrystalline units. This is different from the double diffraction that is a consequence of the dynamical scattering events, for example, the $\:11\overline{1}\:$beam that has $\:{F}_{hkl}\:\ne\:0$ but acts like a new incident beam and the re-diffraction by the $\:\left(1\overline{1}1\right)$ plane results in the 200 reflection^34,35^. Furthermore, the FEM results showed that the forbidden 110 and 200 reflections occur due to the presence of twinning on the 111 planes and that the 111 and 222 reflections appear due to the Friedel’s law^24^. However, this law would not apply to the dynamical electron diffraction but rather apply to kinematical X-ray diffraction. Moreover, the FEM probed region is one order of magnitude larger than that of our NBED observations, which weakens the local diffraction intensities.

## Summary

Proving the absence of a structure is inherently challenging. This concept is known as the devil’s proof. The term “*amorphous*“ is derived from the Latin “*morphe*“ meaning “form,“ prefixed by *a-*, denoting “without.“ Thus, “*amorphous*“ literally means shapeless. This etymology reflects the difficulty in directly demonstrating non-crystallinity of a-Si. In this study, the experimental diffraction patterns of a-Si that is produced by the liquid quenched Ag-Si alloy can be explained by the MD simulations. Furthermore, the NBED observations reveal the appearance of forbidden reflections, even when $\:{F}_{hkl}=0$, indicating a lack of the symmetry in the paracrystals of a-Si. These fundamental insights into the atomic structure of a-Si advance our understanding of amorphous semiconductors^36^, and have significant implications for the atomic scale electrochemical behavior of lithium-^37^ and sodium-^38^ ion batteries with a Si anode.

## Methods

The Ag_79_Si_21_ (at %) master alloy was prepared using pure Ag (99.9%) and pure Si (99.9%) with the arc melting method in an Ar atmosphere. The liquid quenched Ag_79_Si_21_ ribbons were prepared using a single-roll melt-spinning method (NEV-A01, Nisshin Giken Co.). The alloy was melted in a quartz nozzle with an induction heater and the molten alloy was quenched on a Cu roller rotating at 3000 rpm. The diameter of the Cu roller was 200 mm. The ribbon width was about 2 mm and the thickness was about 40 μm.

For the TEM observations, the cross-sectional samples were prepared by a focused ion beam (FIB) equipped with SEM (VERSA 3D, Thermo Fisher Scientific). A ribbon was embedded in an epoxy resin (G2, Gatan) for the sample preparation. A micro-surgical lift-out technique was used during the FIB process and the low acceleration voltage of 8 kV was used to minimize the damage induced by the Ga beam irradiation. The sample thickness is below about 100 nm. For APT observations, needle-shaped samples were prepared by FIB using a dual beam system (Helios nanoLab600i, FEI). Both the TEM and APT samples were obtained from near the wheel side, where the molten alloy directly hit the Cu wheel surface. The structural texture of the wheel side is much finer than that of the other side.

Atomic-scale structural characterization was performed by double spherical aberration corrected STEM with EDS (JEM-ARM200F, JEOL Ltd.). The acceleration voltage was 200 kV and the beam current was 10 pA. The electron diffraction pattern in Fig. 1(f) was obtained with a beam size of ~ 20 nm and a convergence angle of ~ 0.1 mrad. The NBED spots in Fig. 4(c) and (d) were observed during the STEM diffraction imaging mode with a beam size below 1 nm. The EDS analyses were performed to obtain the characteristic X-rays of the elements, and the elemental maps were obtained from the integrated intensity of the X-ray counts. The energy resolution was 128–135 eV at the Mn-Ka line (5.9 keV). The EDS detector was the Si-drift detector with an area size of 30 mm^2^. Chemical analyses with sub-nm resolution were performed using APT (LEAP 4000 XHR, Cameca).

## Data Availability

The data that support the findings of this study are available from the corresponding author upon reasonable request.
